# Anesthetic choice impacts mortality and bacterial clearance in a murine experimental pneumonia model

**DOI:** 10.1186/s12879-025-10785-x

**Published:** 2025-03-27

**Authors:** Hunter Gage, Shawn M. Hannah, Bryan Hancock, Ingrid Cornax, Jason Munguia, Joshua Olson, Elisabet Bjånes, Raymond Zurich, Alexandria Hoffman, Fatemeh Askarian, Khang Tong, Lin Liu, Victor Nizet, Angela Meier

**Affiliations:** 1 Department of Pediatrics, University of California San Diego, La Jolla, CA USA; 2 Altman Clinical and Translational Research Institute, University of California San Diego, La Jolla, CA USA; 3 Herbert Wertheim School of Public Health and Human Longevity Science, University of California, La Jolla, CA USA; 4 Skaggs School of Pharmacy and Pharmaceutical Sciences, University of California, La Jolla, CA USA; 5 Department of Anesthesiology, Division of Critical Care, University of California San Diego, La Jolla, CA USA

**Keywords:** Pneumonia, *P. aeruginosa*, Mouse model, Anesthetics, Ketamine, Isoflurane

## Abstract

**Background:**

Animal models of infectious pneumonia often require the use of anesthetics, but their choice and impact on outcome is rarely discussed. This study investigates the impact of the most commonly used anesthetics on mortality and bacterial clearance in a murine model of *Pseudomonas aeruginosa* pneumonia.

**Methods:**

Isoflurane or ketamine/xylazine were determined to be the most commonly utilized anesthetics for murine pneumonia models. Mice were anesthetized with either ketamine/xylazine or isoflurane during intratracheal infection with *P. aeruginosa* strains PA14 or PA01. Mortality, bacterial clearance, and lung tissue damage were compared. Additional in vitro assays assessed the effects of ketamine on human whole blood killing, serum killing, and neutrophil functions (reactive oxygen species (ROS) production, neutrophil extracellular trap (NET) production, chemotaxis, and phagocytosis).

**Results:**

Mice anesthetized with ketamine/xylazine and infected with PA14 had significantly increased mortality (*p* = 0.004), and significantly higher bacterial burdens in the blood (*p* = 0.01) and lungs (*p* < 0.001). In separate experiments with PA01, mice anesthetized with ketamine/xylazine had significantly increased mortality (*p* = 0.01), higher bacterial burdens in the blood (*p* = 0.01), and higher bacterial burdens in the lungs (*p* = 0.02), along with increased lung tissue pathology (*p* = 0.03) compared to mice anesthetized with isoflurane. Increased mortality and colony forming units were also observed in mice infected under propofol anesthesia, recovered, and subsequently exposed to ketamine versus control (*p* = 0.004 and *p* < 0.001, respectively). Ketamine marginally reduced the killing of PA14 in freshly drawn human whole blood (*p* = 0.0479), but had no significant effect on the serum’s ability to kill PA14. In addition, ketamine reduced in vitro NETosis and chemotaxis (all *p* < 0.05), but had no significant effect on ROS production or phagocytosis of human neutrophils. These in vitro effects were observed only at supraclinical ketamine concentrations.

**Conclusions:**

Our study emphasizes that the choice of anesthetic impacts key outcomes in murine models of pneumonia, and should therefore be an important consideration in experimental design and when comparing results across different studies.

## Introduction

Despite the use of pharmaceutical interventions and public health strategies, lower respiratory tract infections (LRI), including pneumonia, remain the 7th leading cause of death worldwide [1]. Each year, an estimated 344 million cases of LRI occur, leading to approximately 2.18 million deaths [2]. Such public health crises have intensified research efforts to explore the effectiveness of both novel and repurposed therapeutics. Murine models of infection are crucial for studying such therapies and preventive measures. Until recently, the US Food and Drug Administration mandated preclinical testing of all therapeutics in animal models [3]. Although anesthetics are necessary for most experimental murine pneumonia models, their choice and impact is rarely discussed.

Isoflurane and/or a combination of ketamine and xylazine are commonly used anesthetic agents in murine pneumonia models. Previous studies [4–6], including our own [7–9], have investigated the effects of anesthetics on the immune system. For example, isoflurane has demonstrated a protective effect in lung pathology during sterile inflammation. In a murine model employing two-hit lung injury— inhaled lipopolysaccharide (LPS) followed by ventilator-induced lung injury— isoflurane helped preserve tight junctions, maintaining the alveolar-capillary barrier [10]. Additionally, isoflurane inhibited LPS-induced lung injury and neutrophil infiltration [11]. A study using various inhalational anesthetics in a murine model of ventilator-induced lung injury showed certain protective effects mediated through anti-inflammatory and antioxidant properties [12]. Recent research also revealed that isoflurane attenuates sepsis-associated lung injury in a murine model [13].

Ketamine has been shown to reduce lung pathology in a rat model of LPS-induced lung injury [6] and to decrease airway inflammation in a rat model of asthma [14]. Despite the widespread use of different anesthetics in murine models, no studies have directly compared their impact on outcomes in infectious models using a precise intratracheal infection challenge.

In this study, we demonstrate the differential effect of ketamine and isoflurane on key outcomes of bacterial pneumonia in a murine model of *Pseudomonas aeruginosa* infection induced by direct tracheal instillation. We illustrate and emphasize that the choice of anesthetic is an important, yet often overlooked, consideration when comparing findings reported in experimental models of bacterial pneumonia. Additionally, in vitro experiments with ketamine and human neutrophils suggest a possible modulatory role of ketamine on neutrophil function.

## Material and methods

### Bacteria

Wild-type (WT) human isolated *P. aeruginosa* strains PA14 (UCBPP-PA14) [15] (previously provided by Prof. Nina van Sorge, University of Amsterdam, NL) and PA01 [16] (previously provided by Prof. Colin Manoil, University of Washington, USA) were grown overnight in Luria–Bertani (LB) broth at 37 °C in a shaking incubator. A subculture was grown to logarithmic phase (OD_600_ ~ 0.6), washed with phosphate-buffered saline (PBS), resuspended to an OD_600_ ~ 0.4, and then diluted to the desired concentration in either PBS or RPMI. For in vivo experiments, the inoculum was serial diluted and plated for accuracy, with colony forming units (CFU) counted the following day. In some experiments involving strain PA01, bacteria were frozen at log phase, stored at −80 °C, and thawed on the day of infection.

### Mice

Female CD-1 mice, aged 7–13 weeks (Charles River Labs, strain 022), were housed in a specific pathogen-free vivarium under a 12 h light/dark cycle with ad libitum access to food and water. All mouse experiments were conducted in accordance with the guidelines of the federal Animal Welfare Act and were approved by the University of California San Diego (UCSD) Institutional Animal Care and Use Committee (IACUC; protocol #S00227M).

### Intratracheal infection model

Mice were intratracheally infected with *Pseudomonas* under two different anesthetic regimens: 1) ten minutes of inhaled isoflurane, or 2) a combination of ketamine (100 mg/kg) and xylazine (10 mg/kg) administered intraperitoneally (IP). In separate experiments, mice were given IV propofol (20 mg/kg) into the tail vein, infected intratracheally, allowed to recover, and then administered IP ketamine (100 mg/kg) or PBS control. For the intratracheal infections, an otoscope was used to directly visualize the vocal cords. A micropipette equipped with a gel loading tip was passed through the otoscope’s speculum to deliver the inoculum intratracheally onto the vocal cords. Mice were infected with 30 μL of 10^6^ to 10^7^ CFUs of PA14 or PA01, diluted in PBS. Post-infection, mice recovered on a warming pad and were monitored for survival. To assess the bacterial burden, mice were euthanized by CO_2_ asphyxiation approximately 25 h post-infection (PA14) and approximately 12–24 h post-infection (PA01). Blood was drawn by cardiac puncture, mixed with heparin, plated on LB agar, and CFUs enumerated the following day. In cases where blood CFUs were too high to count, the CFU value from the mouse with the highest enumerable bacterial burden was defined as the upper limit of quantification. To determine CFU counts per gram of lung tissue, lungs were isolated, weighed, homogenized in a morcellator with beads, serially diluted, and plated on LB agar.

Bronchoalveolar fluid (BAL) was collected after mice were euthanized by CO_2_ asphyxiation the day after infection. Lungs were flushed with 1 mL of 0.5 mM EDTA + PBS, this was serially diluted, and plated on LB agar to enumerate CFUs. In cases where both CFU/g lung tissue and histology were performed on the same mice, one lung was used for each analysis. One lung was removed for CFU count (as above) and the remaining lung was flushed with 1 mL of zinc-buffered formalin, removed from the thoracic cavity, routinely processed, and embedded in paraffin. Four μm lung sections were stained with hematoxylin and eosin (H&E) and examined by a board-certified veterinary pathologist (IC), blinded to the treatment conditions, to assess the distribution and severity of tissue damage and leukocyte infiltration. The sections were blindly scored for overall severity using a six-grade system: 0 indicates no significant findings; 1–5 indicate minimal, mild, moderate, marked, and severe inflammation, respectively. Minimal and mild severities were characterized by low levels of neutrophilic inflammation without necrosis or disruption of normal pulmonary architecture. Sections from moderately to severely affected lungs showed increasing degrees of tissue necrosis, neutrophilic toxic change, and loss of alveolar wall integrity, evidenced by the presence of proteinaceous fluid (edema) and fibrin threads within air spaces (alveoli and terminal bronchi).

### Human neutrophil and whole blood isolation

Our studies with human neutrophils and whole blood were conducted with approval from the UCSD Institutional Review Board (IRB, #131002). Blood was drawn from consenting healthy volunteers into 10 mL heparin tubes (BD Biosciences). Whole blood was used directly, while neutrophils were isolated using Polymorphprep (Serumwerk Bernburg AG for Alere Technologies, AS, Oslo, Norway) as described elsewhere [9]. Ketamine concentration ranges applied in subsequent in vitro experiments mirrored those achievable in plasma during general anesthesia in humans [17], as well as concentrations 10X and 100X higher, ranging from 3 µg/mL to 300 µg/mL.

### Reactive oxygen species (ROS) measurement

The ability of freshly isolated human neutrophils to produce ROS was evaluated as described elsewhere [18]. Briefly, neutrophils were diluted to 2 × 10^6^ cells/mL in HBSS-/- and incubated with a 10 µM solution of 2',7'-dichlorodihydrofluorescein diacetate (DCFDH) for 20 min. Labeled neutrophils were then seeded in triplicate into 96-well plates at a concentration of 1 × 10^6^ cells/mL in HBSS + / + and stimulated with phorbol 12-myristate 13-acetate (PMA), in the presence or absence of ketamine at indicated concentrations. ROS production was monitored by reading fluorescence (485 nm excitation, 530 nm emission) using an EnVision plate reader (Perkin Elmer). Measurements were taken at specified intervals over 2 h, maintaining the plate at 37 °C and 5% CO_2_, shielded from light between readings.

### Neutrophil extracellular trap (NET) production

The capacity of human neutrophils to produce extracellular traps was assessed in a 96-well plate in HBSS + / + using PicoGreen (Thermo Fisher) as described elsewhere [9]. Neutrophils, freshly isolated and diluted to 500,000 cells per well in triplicate, were preincubated with either ketamine or a control solution at the indicated concentrations for 30 min at 37 °C and 5% CO_2_. After preincubation, 25 nM PMA was added, and the plate was incubated for a further 2 h under the same conditions. NETs were digested by adding micrococcal nuclease for 10 min, then the reaction was halted with EDTA. PicoGreen was subsequently added per manufacturer’s instructions, and NET production was quantified using an EnVision Alpha plate reader (Perkin Elmer; 480 nm excitation, 520 nm emission).

### Neutrophil phagocytosis

The phagocytic activity of human neutrophils in the presence or absence of ketamine was assessed using pHrodo Red *E. coli* BioParticles (Invitrogen) following the manufacturer’s guidelines. Neutrophils were isolated, resuspended to 1 × 10^7^ cells/mL, and preincubated with indicated concentrations of ketamine for 30 min. The cells (2 × 10^5^ per well), along with *E. coli* BioParticles, were then transferred to a 96-well plate in triplicate. Fluorescence was measured at 560/585 nm (excitation/emission) every 30 min for 2 h. Plates were covered with foil to protect from light and maintained at 37 °C with 5% CO_2_ between measurements.

### Neutrophil chemotaxis

The influence of ketamine on human neutrophil chemotaxis was determined using a transwell system with 3.0 µm polycarbonate membranes (Corning). In brief, 1 × 10^6^ isolated human neutrophils were preincubated for 30 min with either ketamine or PBS as a control, at specified concentrations in triplicate. These cells were then placed in the upper compartment of the transwell setup. Matching concentrations of ketamine, as well as FMLP (Sigma) or HBSS + / + , were added to the lower compartments. The chambers were incubated for 45 min at 37 °C and 5% CO_2_. Post-incubation, the number of neutrophils that had migrated to the lower compartment was quantified with p-nitroanilide (Sigma) to measure neutrophil elastase activity. Cells in the bottom well were lysed with 1% Triton X-100 in PBS for 10 min at room temperature. A final concentration of 10 mM p-nitroanilide (1 mM in mixture) was added and the samples were incubated at room temperature for 30 min. Absorbance was measured at 405 nm.

### Whole blood killing

To investigate the effect of ketamine on the bactericidal activity of whole blood against PA14, blood drawn from healthy volunteers into heparin tubes was aliquoted into siliconized tubes. Blood was pre-incubated with either ketamine (3–30 μg/mL) or RPMI (as a control) in triplicate for 15 min on a rotator at 37 °C. PA14 was added at ~ 1.5 × 10^6^ CFUs and tubes were then incubated for 30 min on a rotator at 37 °C. Subsequently, ice-cold 0.3% saponin was added to each tube and incubated on ice for 5 min to lyse the phagocytes. Samples were serially diluted in PBS and plated on LB agar for enumeration the next day. Percent killing was calculated by comparing the CFUs to the initial inoculum.

### Serum killing

To assess the effect of ketamine on the bactericidal activity of serum, previously pooled serum from six donors (3 male, 3 female) stored at −80 °C was thawed and aliquoted into siliconized tubes in triplicate. Pooled serum was pre-incubated with ketamine (3–300 μg/mL) or RPMI (as a control) on a rotator at 37 °C for 15 min. PA14 was added at ~ 1.5 × 10^6^ CFUs (bringing the final dilution of serum after the addition of bacteria to 1:10). The tubes were then incubated on a rotator at 37 °C for 75 min. Following incubation, ice-cold 0.3% saponin was added to each tube, incubated on ice for 5 min, then serially diluted in PBS and plated on LB agar for enumeration the next day. Percent killing was calculated by comparing the CFUs to the initial inoculum.

### Statistics

Statistical analyses and graphical representations were performed using GraphPad Prism 10. For highly skewed data, CFUs were log10-transformed before analysis. Mann–Whitney tests were used to compare two groups. For comparisons among multiple groups, Kruskal–Wallis tests were conducted, accompanied by pairwise comparisons and Dunn’s post-test corrections. These more robust, nonparametric tests were chosen to account for smaller sample sizes. Kaplan–Meier curves between groups were compared using a log-rank test. A *p*-value < 0.05 was considered statistically significant. All selected statistical methods were validated through consultation with statisticians from the UCSD Altman Clinical and Translational Research Institute (ACTRI).

## Results

### Isoflurane and ketamine are the most commonly used anesthetics for murine bacterial pneumonia models

**Fig. 1:**
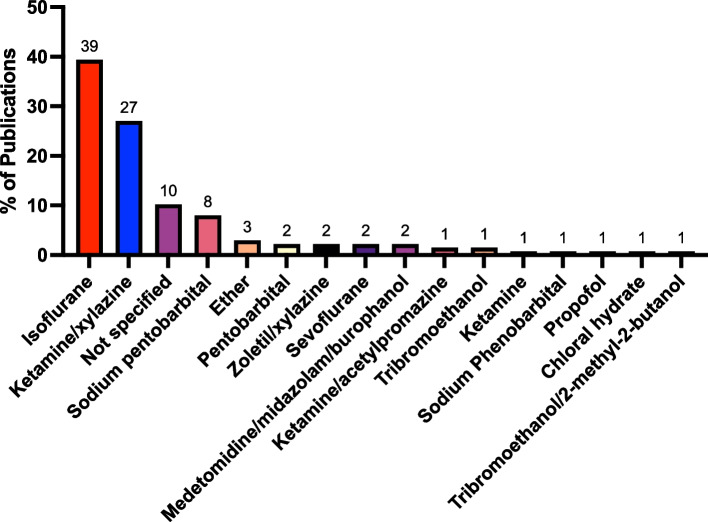
Prevalence of different anesthetics in mouse models of infectious pneumonia and those involving LPS in studies published from Nov. 28th, 2022 to Nov. 28th, 2023

We conducted a literature search using PubMed on November 28th, 2023, with the keywords “bacterial pneumonia, mouse,” limiting the results to publications from the previous year. This search included all models of infectious pneumonia and those using LPS. We excluded studies involving non-bacterial or non-viral infection models, a drinking water model that did not result in infection, and one study that was not accessible. We also included studies published with BioRx (2). Of 190 studies analyzed, 137 described the use of anesthetics (Fig. 1). Isoflurane was the most commonly used anesthetic, employed in 39.4% of studies, followed by a combination of ketamine and xylazine in 27% of the studies. An additional 10.2% used an anesthetic but did not specify which one, while sodium pentobarbital was used in 8% of the cases. Other less frequently used anesthetics are detailed in Fig. 1.

### Ketamine/xylazine anesthesia reduces survival and increases bacterial burden in *P. aeruginosa* pneumonia compared to isoflurane

**Fig. 2:**
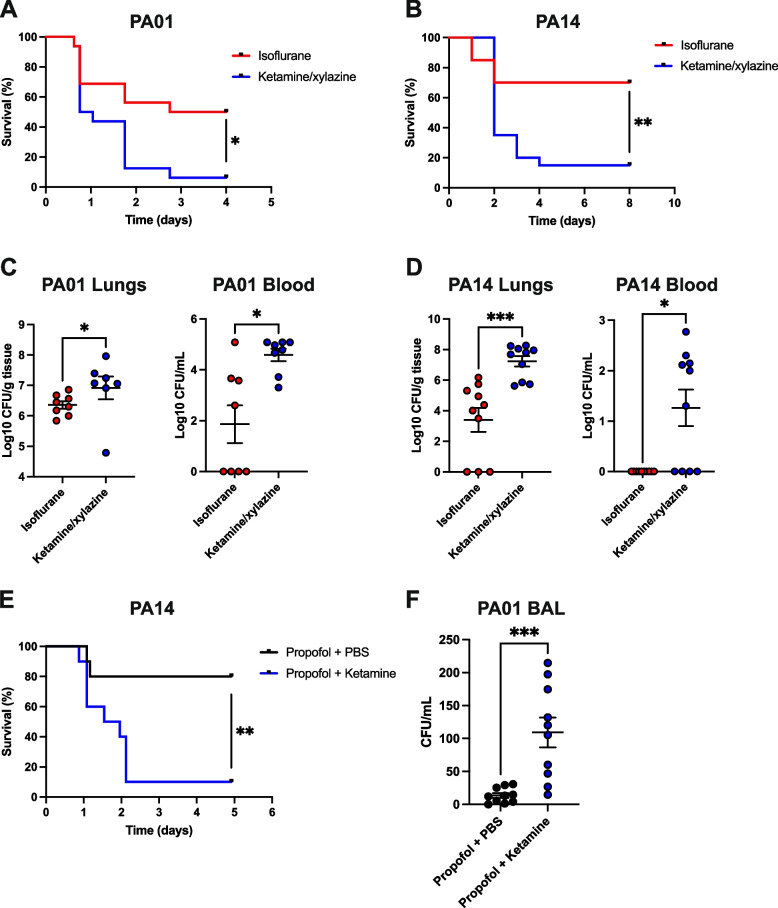
**A** and **B** Kaplan–Meier curves of CD-1 mice anesthetized with isoflurane or ketamine/xylazine and infected IT with PA01 or PA14, respectively (*n* = 16 per group for PA01, *n* = 20 per group for PA14, data from two independent experiments each). **C** and **D** Log-CFUs in the lungs (left panels) and blood (right panels) in mice anesthetized with isoflurane or ketamine/xylazine and infected IT with PA01 or PA14, respectively (*n* = 8 per group for PA01, *n* = 10 per group for PA14). For blood CFUs in mice infected with PA01, four samples had a bacterial load that was too numerous to count. In these cases, the CFU value from the mouse with the highest enumerable bacterial burden was used as the upper limit of quantification (log10CFU/mL = 5.08). **E** Kaplan–Meier curve and **F** CFUs in bronchoalveolar lavage fluid from CD-1 mice anesthetized with propofol, infected IT with PA14 or PA01, allowed to recover, and treated with ketamine or PBS control (*n* = 10 per group, data from one experiment). Data are represented as mean ± SEM. *P*-values for Kaplan–Meier curves were calculated by log-rank tests. *P*-values for CFU comparisons were calculated by Mann–Whitney U tests. *p < 0.05, **p < 0.01, ***p < 0.001

To evaluate the impact of anesthetic choice on survival in a murine model of pneumonia, we anesthetized mice with either isoflurane or ketamine/xylazine and performed intratracheal infections with *P. aeruginosa* strains PA01 or PA14. Mice infected with PA01 and anesthetized with isoflurane had a significantly higher percent survival rate than those that received ketamine/xylazine (Fig. 2A, *p* = 0.01). This result was replicated in separate experiments in which mice were infected with PA14 (Fig. 2B, *p* = 0.004).

We also assessed the bacterial load following intratracheal infection under ketamine/xylazine versus isoflurane anesthesia using the two different *P. aeruginosa* strains. Mice infected with PA01 and anesthetized with ketamine/xylazine had a higher (five-fold) median bacterial CFU count in their lungs (Fig. 2C, *p* = 0.02) and a higher (20-fold) median bacterial CFU count in their blood (Fig. 2C, *p* = 0.01), compared to those anesthetized with isoflurane. In a separate experiment with PA14, mice under ketamine/xylazine anesthesia exhibited over a 3000-fold higher median bacterial CFU count in the lungs (Fig. 2D, *p* < 0.001) than those receiving isoflurane. Additionally, mice anesthetized with isoflurane had undetectable levels of PA14 in the blood, whereas most ketamine/xylazine-anesthetized mice showed bacteremia (Fig. 2D, *p* = 0.01).

### I.p. ketamine lowers survival and increases CFU count in mice infected with *P. aeruginosa* under propofol anesthesia when administered after infection

To exclude the possibility that different distribution of bacteria could be mediated by distinct breathing patterns specific to either ketamine or isoflurane, mice were infected intratracheally with *Pseudomonas* under propofol anesthesia. After recovery from the propofol anesthesia, either i.p. ketamine or i.p. PBS was administered and survival and CFU analyses were performed as above. As compared to PBS controls, mice given i.p. ketamine following *P. aeruginosa* infection had significantly higher mortality (Fig. 2E, *p* = 0.004, strain PA14) and eight-fold higher median CFU counts in BAL (Fig. 2F, *p* < 0.001, strain PA01) the day after infection.

### Ketamine/Xylazine anesthesia is associated with increased tissue necrosis in *P. aeruginosa* pneumonia when compared to isoflurane anesthesia

**Fig. 3:**
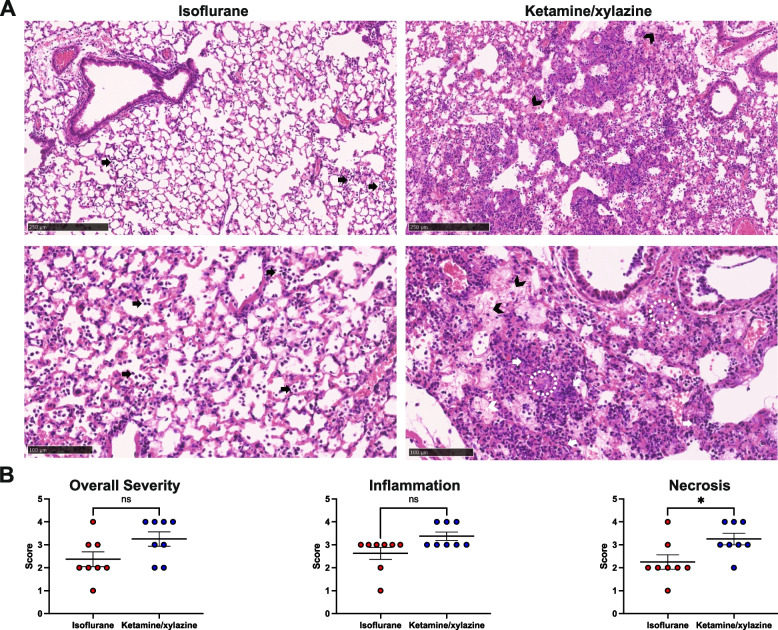
**A** H&E-stained 4-µm thick sections of lungs from mice anesthetized with isoflurane (left panels) or ketamine/xylazine (right panels) and infected IT with PA01. Black arrows indicate scattered neutrophilic infiltrates into air spaces. White dashed ovals denote air spaces filled with inflammatory exudate including visible bacterial colonies. Black chevrons indicate fibrinous pulmonary edema. White arrows indicate neutrophils with toxic changes and nuclear streaming. **B** Lung section scores for overall severity, inflammation, and necrosis using a six-grade system, where higher values indicate more severe findings (*n* = 8 per group, data from one experiment). *P*-values were calculated by Mann–Whitney U tests. NS = not significant, *p < 0.05

H&E-stained 4-μm-thick lung sections from mice inoculated intratracheally with PA01 were examined. In the lungs of mice treated with isoflurane and infected with PA01, inflammatory changes were generally mild and consisted of scattered neutrophilic infiltrates into air spaces (Fig. 3A). In the ketamine/xylazine-treated group, the inflammatory findings were more severe. Air spaces were filled with inflammatory exudate including visible bacterial colonies, fibrinous pulmonary edema, and large numbers of neutrophils, commonly displaying toxic changes and nuclear streaming. In mice receiving ketamine/xylazine anesthesia, the incidence and severity of tissue damage, necrosis, and loss of alveolar wall integrity were significantly higher compared to those anesthetized with isoflurane (Fig. 3B, *p* = 0.03).

### Ketamine impacts human whole blood and neutrophil function in vitro

**Fig. 4:**
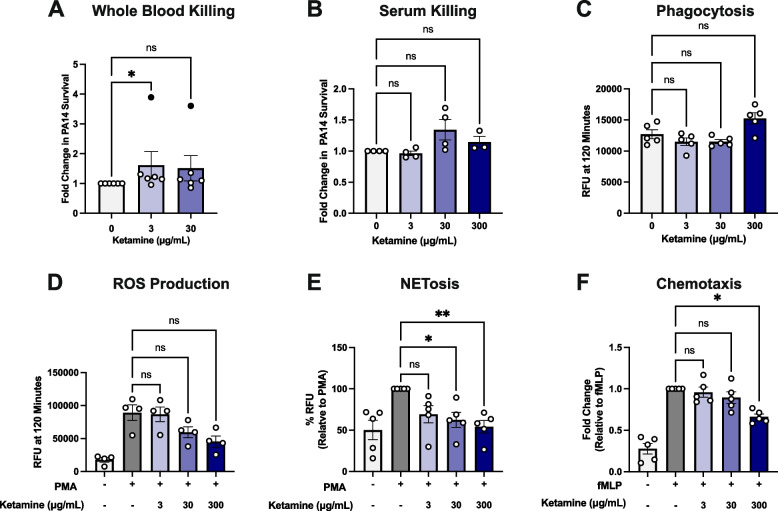
**A** Fold change in PA14 survival when incubated with human whole blood and treated with ketamine, relative to PBS control (*n* = 6 independent individual donors per group). One donor in the 3 µg/mL and 30 µg/mL ketamine groups was an outlier (data points are indicated by filled black circles). There are no statistically significant differences between groups if the outlier is excluded. **B** Fold change in PA14 survival when incubated with human serum and treated with ketamine, relative to PBS control (*n* = 3–4 experiments with same pooled serum per group). **C** Phagocytosis of E. coli bioparticles at 120 min by human neutrophils treated with ketamine (*n* = 5 individual donors per group). **D** ROS production at 120 min in human neutrophils stimulated by PMA and treated with ketamine (*n* = 4 individual donors per group). **E** NETosis in human neutrophils treated with ketamine and stimulated by PMA for 3 h, relative to PBS control (*n* = 5 individual donors per group). **F** Chemotaxis of human neutrophils stimulated by fMLP and treated with ketamine, relative to PBS control (*n* = 5 individual donors per group). Data are represented as the average of technical replicates per donor ± SEM. *P*-values were calculated by Kruskal–Wallis tests with Dunn’s post-test corrections. NS = not significant, *p < 0.05, **p < 0.01

To examine the direct effect that ketamine might have on the ability of human whole blood or serum to kill *P. aeruginosa*, we performed in vitro whole blood killing assays with blood from healthy human volunteers and serum killing assays with pooled human serum. Treatment with 3 µg/mL ketamine resulted in higher survival of PA14 in whole blood (Fig. 4A, *p* = 0.048), though this result was not statistically significant if data points from an outlier donor were excluded. At a concentration of 30 µg/mL ketamine, PA14 survival trended higher, though this difference was not statistically significant. In human serum, ketamine did not affect PA14 killing at any tested concentrations (Fig. 4B, *p* > 0.05).

Because neutrophils play a key role in defending against bacterial pneumonia, we investigated whether ketamine might directly affect neutrophil functions. We conducted a series of in vitro experiments with freshly isolated human neutrophils. While ROS production and phagocytosis were not affected by any concentrations of ketamine tested, ketamine reduced neutrophil extracellular traps (NETs) at concentrations of 30 µg/mL and 300 µg/mL, compared to PMA control (Fig. 4C-E, *p* = 0.03 and *p* = 0.0049, respectively). At 300 µg/mL, ketamine reduced chemotaxis compared to fMLP control (Fig. 4F, *p* = 0.04).

## Discussion

Despite the known effects of anesthetics on the immune system [19], their differential impact on the course of infectious pneumonia remains underexplored. While the use of anesthetics in murine models of pneumonia is widespread, the impact of anesthetics on outcomes in these models is not routinely considered.

Our literature search revealed that isoflurane and ketamine are the most commonly used anesthetics for models of murine infectious pneumonia. We observed increased mortality and bacterial burden with ketamine/xylazine versus isoflurane anesthesia in an IT murine model of pneumonia. Differential respiratory physiology, such as breath holding with isoflurane, might influence initial uptake, as demonstrated by Seo et al. [20], who showed differences in the distribution of intranasally administered ^125^I-albumin depending on whether ketamine/xylazine or isoflurane was used. However, our intratracheal infection method under direct visualization of the vocal cords ensured that significant reflux did not occur. Moreover, this detrimental impact of ketamine was replicated when animals were infected under propofol anesthesia, recovered, and later administered i.p. ketamine, suggesting that the observed effects were not due to differences in initial uptake pattern but rather due to the differential impact of anesthetics on the immune system. While anesthesia recovery time and animal body temperature was not systematically recorded in our study, it is possible that body temperature despite warming efforts differed between groups which in turn may have affected immune responses. Other indirect effects of anesthetics on the immune response may be mediated through their differential impact on the autonomic nervous system, which is known to regulate immune cellular responses [21].

The impact of anesthetics on lung inflammation has been studied in response to LPS, but direct comparisons in bacterial pneumonia models are limited. Our findings indicate that ketamine had a detrimental effect on the course of pneumonia in murine studies. While ketamine is known to diminish immune responses in LPS-induced lung injury [6], these anti-inflammatory effects may be detrimental in the context of an infection. Recent studies have shown that hypoxia-induced antibacterial defenses are inhibited by ketamine, in vitro and in vivo [6, 22, 23], indicating that, while inflammation can be reduced, immune defenses may be compromised. Our study demonstrated significantly increased tissue necrosis in the presence of ketamine during pseudomonas infection. Given the known anti-inflammatory effects of ketamine in the absence of infection [24], we attributed the observed increase in necrosis to the enhanced infectious spread and not to any direct effect of ketamine/xylazine alone.

Our in vitro experiments on human neutrophil functions revealed that ketamine did not significantly inhibit isolated phagocytosis or ROS production. However, at higher concentrations, ketamine did inhibit neutrophil NETosis and chemotaxis. Overall, at concentrations of ketamine reported to be achieved in the serum of anesthetized humans [17], there was no significant in vitro impairment of the immune functions we investigated that would fully explain our in vivo findings. This further emphasizes the possibility of an indirect effect of ketamine on the immune response as an interesting aspect of future investigation.

## Conclusions

Our in vivo murine experiments indicate that the choice of anesthetic can significantly impact the course of bacterial pneumonia in mice. The detrimental effect of ketamine remained apparent even when administered after the infection. Ketamine did not significantly inhibit human neutrophil function in vitro at concentrations achieved during anesthesia in humans. These results highlight a previously unrecognized confounding factor when directly comparing experimental models of murine pneumonia, suggesting that the choice of anesthetic could influence the perception—up or down—of the virulence of a bacterial pathogen, function of a host immune pathway, or efficacy of a therapeutic intervention. Choice of anesthetics should be carefully considered when interpreting data reported in preclinical studies that employ animal infection models, as well as in the design of future such studies.

Isoflurane and ketamine, which differentially impacted mortality, bacterial clearance, and inflammatory responses in our murine experimental pneumonia model, are widely used for surgical anesthesia and for sedation in intensive care unit patients, including those receiving mechanical ventilation. Future observational studies in human clinical cohorts to assess whether specific anesthetic agents influence the incidence, severity, or outcome of bacterial pneumonia in at-risk patients may merit consideration.

## Data Availability

The data underlying this article will be shared upon reasonable request to the corresponding author.

## Abbreviations

LRI: Lower respiratory tract infection
LPS: Lipopolysaccharide
LB: Luria–Bertani
PBS: Phosphate-buffered saline
CFU: Colony forming unit
IP: Intraperitoneally
IV: Intravenously
BAL: Bronchoalveolar lavage
H&E: Hematoxylin and eosin
ROS: Reactive oxygen species
HBSS-/-: Hank’s balanced salt solution (without Ca^2+^ and Mg^2+^)
HBSS + / +: Hank’s balanced salt solution (with Ca^2+^ and Mg^2+^)
fMLP: N-formyl-methionyl-leucyl-phenylalanine
PMA: Phorbol 12-myristate 13-acetate
NET: Neutrophil extracellular trap
MOI: Multiplicity of infection
ANOVA: Analysis of variance
RFU: Relative fluorescence units
